# *In Situ* Forming Polymeric Drug Delivery Systems

**DOI:** 10.4103/0250-474X.56015

**Published:** 2009

**Authors:** M. Madan, A. Bajaj, S. Lewis, N. Udupa, J. A. Baig

**Affiliations:** C. U. Shah College of Pharmacy, S. N. D.T. Women's University, Mumbai-400 049, India; 1 Manipal College of Pharmaceutical Sciences, Manipal University, Manipal-576 104, India; 2 Faculty of Pharmacy, Jamia Hamdard, New Delhi-110 062, India

**Keywords:** Biodegradable polymers, controlled release, *in situ* gels, poly (lactic-co-glycolic acid), sustained release

## Abstract

*In situ* forming polymeric formulations are drug delivery systems that are in sol form before administration in the body, but once administered, undergo gelation *in situ*, to form a gel. The formation of gels depends on factors like temperature modulation, pH change, presence of ions and ultra violet irradiation, from which the drug gets released in a sustained and controlled manner. Various polymers that are used for the formulation of *in situ* gels include gellan gum, alginic acid, xyloglucan, pectin, chitosan, poly(DL-lactic acid), poly(DL-lactide-co-glycolide) and poly-caprolactone. The choice of solvents like water, dimethylsulphoxide, N-methyl pyrrolidone, triacetin and 2-pyrrolidone for these formulations depends on the solubility of polymer used. Mainly *in situ* gels are administered by oral, ocular, rectal, vaginal, injectable and intraperitoneal routes. The *in situ* gel forming polymeric formulations offer several advantages like sustained and prolonged action in comparison to conventional drug delivery systems. The article presents a detailed review of these types of polymeric systems, their evaluation, advancements and their commercial formulations. From a manufacturing point of view, the production of such devices is less complex and thus lowers the investment and manufacturing cost.

Extensive research has been carried in designing of polymeric drug delivery systems. The development of *in situ* gel systems has received considerable attention over the past few years[1]. This interest has been sparked by the advantages shown by *in situ* forming polymeric delivery systems such as ease of administration and reduced frequency of administration, improved patient compliance and comfort[2].

In the past few years, increasing number of *in situ* gel forming systems have been investigated and many patents for their use in various biomedical applications including drug delivery have been reported. Smart polymeric systems represent promising means of delivering the drugs; these polymers undergo sol-gel transition, once administered[3]. *In situ* gel formation occurs due to one or combination of different stimuli like pH change, temperature modulation and solvent exchange. In this review, different types of smart polymers, their mechanisms of gel formation from the sol forms, evaluation and characterization of *in situ* polymeric formulations are discussed. From the early 1970's natural and synthetic polymers began to be investigated for controlled release formulations. The advantages of using biodegradable polymers in clinical applications are apparent. Various natural and synthetic polymers are used for formulation development of *in situ* forming drug delivery systems. Depending on the route of administration, these *in situ* polymeric systems may be classified as illustrated in following sections.

## *IN SITU* FORMING POLYMERIC SYSTEMS FOR ORAL ADMINISTRATION

Pectin, xyloglucan and gellan gum are the natural polymers used for *in situ* forming oral drug delivery systems. Pectins are a family of polysaccharides, in which the polymer backbone mainly comprises α-(1-4)-D-galacturonic acid residues[4]. Low methoxypectins (degree of esterification <50%) readily form gels in aqueous solution in the presence of free calcium ions, which crosslink the galacturonic acid chains in a manner described by egg-box model[5]. Although the gelation of pectin will occur in the presence of H^+^ ions, a source of divalent ions, generally calcium ions is required to produce the gels that are suitable as vehicles for drug delivery. The potential of an orally administered *in situ* gelling pectin formulation for the sustained delivery of paracetamol has been reported[4]. The main advantage of using pectin for these formulations is that it is water soluble, so organic solvents are not necessary in the formulation. Divalent cations present in the stomach, carry out the transition of pectin to gel state when it is administered orally[6]. Calcium ions in the complexed form may be included in the formulation for the induction of pectin gelation.

Sodium citrate may be added to the pectin solution to form a complex with most of calcium ions added in the formulation. By this means, the formulation may be maintained in a fluid state (sol), until the breakdown of the complex in the acidic environment of the stomach, where release of calcium ions causes gelation to occur. The quantities of calcium and citrate ions may be optimized to maintain the fluidity of the formulation before administration and resulting in gelation, when the formulation is administered in the stomach.

Xyloglucan is a polysaccharide derived from tamarind seeds and is composed of a (1-4)-β-D-glucan backbone chain, which has (1-6)-α-D xylose branches that are partially substituted by (1-2)-β-D-galactoxylose[7]. When xyloglucan is partially degraded by β-galactosidase, the resultant product exhibits thermally reversible gelation by the lateral stacking of the rod like chains[7]. The sol-gel transition temperature varies with the degree of galactose elimination. Xyloglucan gels have potentially been used for oral, intraperitoneal, ocular and rectal drug delivery[8–11]. It forms thermally reversible gels on warming to body temperature. Its potential application in oral delivery exploits the proposed slow gelation time (several minutes) that would allow *in situ* gelation in the stomach following the oral administration of chilled xyloglucan solution. The gelation behaviour of xyloglucan is similar to that observed with Pluronic F127, with a sol-gel transition on heating from refrigerator temperature or cooling from a higher temperature[12]. But the important difference between the gelation properties of the xyloglucan and Pluronic F127 from a formulation view point is that xyloglucan forms gels at much lower concentration[11]. The use of xyloglucan as a polymer for drug delivery through other routes of administration is discussed later.

Gellan gum (commercially available as Gelrite™ or Kelcogel™) is an anionic deacetylated exocellular polysaccharide secreted by *Pseudomonas elodea* with a tetrasaccharide repeating unit of one α-L-rhamnose, one β-D-glucuronic acid and two β-D-glucuronic acid residues[13]. It has the tendency of gelation which is temperature dependent or cations induced[14]. This gelation involves the formation of double helical junction zones followed by aggregation of the double helical segments to form a three-dimensional network by complexation with cations and hydrogen bonding with water[15–17]. *In situ* gelling gellan formulation as vehicle for oral delivery of theophylline is reported[13]. The formulation consisted of gellan solution with calcium chloride and sodium citrate complex. When administered orally, the calcium ions are released in acidic environment of stomach leading to gelation of gellan thus forming a gel *in situ*. An increased bioavailability with sustained drug release profile of theophylline in rats and rabbits was observed from gellan formulations as compared to the commercial sustained release liquid dosage form.

## *IN SITU* FORMING POLYMERIC SYSTEMS FOR OCULAR DELIVERY

For *in situ* gels based ocular delivery, natural polymers such as gellan gum, alginic acid and xyloglucan are most commonly used polymers. Local ophthalmic drug delivery has been used for various compounds such as antimicrobial agents, antiinflammatory agents and autonomic drugs used to relieve intraocular tension in glaucoma. Conventional delivery systems often result in poor bioavailability and therapeutic response because high tear fluids turn over and dynamics cause rapid elimination of the drug from the eyes[18,19]. So, to overcome bioavailability problems, ophthalmic *in situ* gels were developed[20]. Aqueous solution of gellan dropped into the eye undergoes transition into the gel state due to the temperature and ionic condition (Ca^++^) in the tear fluid[21]. Much of the interest in the pharmaceutical application of gellan gum has concentrated on its application for ophthalmic drug delivery[22–24]. Drug release from these *in situ* gels is prolonged due to longer precorneal contact times of the viscous gels compared with conventional eye drops.

Alginic acid is a linear block copolymer polysaccharide consisting of β-D-mannuronic acid and α-L-glucuronic acid residues joined by 1,4-glycosidic linkages[7,25]. The proportion of each block and the arrangement of blocks along the molecule vary depending on the algal source. Dilute aqueous solutions of alginates form firm gels on addition of di- and trivalent metal ions by a cooperative process involving consecutive glucuronic residues in the α-L-glucuronic acid blocks of the alginate chain. Alginic acid can be chosen as a vehicle for ophthalmic formulations, since it exhibits favorable biological properties such as biodegradability and nontoxicity[26]. A prolonged precorneal residence of formulations containing alginic acid was looked for, not only based on its ability to gel in the eye, but also because of its mucoadhesive properties[27,28].

Miyazaki *et al.* attempted to formulate *in situ* gels for ocular delivery using xyloglucan (1.5% w/w) as the natural polymer[10]. These *in situ* forming polymeric systems were observed to show a significant mitotic response for a period of 4h when instilled into lower cul-de-sac of rabbit eye.

Various water soluble polymers such as carbopol system- hydroxypropylmethylcellulose system, poly (methacrylic acid)-poly (ethylene glycol) come under the category of pH-induced *in situ* precipitating polymeric systems. Carbopol is a well known pH dependent polymer, which stays in solution form at acidic pH but forms a low viscosity gel at alkaline pH. HPMC is used in combination with carbopol to impart the viscosity to carbopol solution, while reducing the acidity of the solution[29]. Based on this concept, the formulation and evaluation of an ophthalmic delivery system for indomethacin for the treatment of uveitis was carried out. A sustained release of indomethacin was observed for a period of 8 h *in vitro* thus considering this system as an excellent candidate for ocular delivery.

## *IN SITU* FORMING POLYMERIC SYSTEMS FOR RECTAL AND VAGINAL DELIVERY

*In situ* gels also possess a potential application for drug delivery by rectal and vaginal route. Miyazaki *et al.* investigated the use of xyloglucan based thermoreversible gels for rectal drug delivery of indomethacin[11]. Administration of indomethacin loaded xyloglucan based systems to rabbits indicated broad drug absorption peak and a longer drug residence time as compared to that resulting after the administration of commercial suppository. In addition, a significant reduction of drug C_max_ was observed after administration of *in situ* polymeric system thus indicating the avoidance of adverse effects of indomethacin on nervous system.

For a better therapeutic efficacy and patient compliance, a mucoadhesive, thermosensitive, prolonged release vaginal gel incorporating clotrimazole-β-cyclodextrin complex was formulated for the treatment of vaginitis[12]. Pluronic F-127 was used as an *in situ* gel forming polymer together with mucoadhesive polymers such as Carbopol 934 and hydroxylpropylmethylcellulose to ensure long residence time at the application site. Controlled release of drug was achieved *in vitro* indicating antimycotic efficacy of developed formulation for a longer period of time.

## *IN SITU* FORMING INJECTABLE DRUG DELIVERY SYSTEMS

The development of injectable *in situ* forming drug delivery systems has received a considerable interest over the last decade. Chitosan is a biodegradable, thermosensitive, polycationic polymer obtained by alkaline deacetylation of chitin, a natural component of shrimp and crab shell[30]. Chitosan is a biocompatible pH dependent cationic polymer, which remains dissolved in aqueous solutions up to a pH of 6.2. Neutralization of chitosan aqueous solution to a pH exceeding 6.2 leads to the formation of a hydrated gel like precipitate. The main problem with chitosan is its non-biodegradability[30]. The pH gelling cationic polysaccharides solution are transformed into thermally sensitive pH dependent gel forming aqueous solutions, without any chemical modification or cross linking by addition of polyol salts bearing a single anionic head such as glycerol, sorbitol, fructose or glucose phosphate salts to chitosan aqueous solution. This transformation has solved the non-biodegradability problem of chitosan. This type of thermosensitive gel formulation was tried by Chenite *et al*., where the formulation was in the SOL form at room temperature, in which living cells and therapeutic proteins can be incorporated[31]. This formulation, when injected *in vivo*, turns into gel implants *in situ.* This system was used successfully to deliver biologically active growth factors *in vivo* as well as an encapsulated matrix for living chondrocytes for tissue engineering applications.

A novel, injectable, thermosensitive *in situ* gelling hydrogel was developed for tumor treatment. This hydrogel consisted of drug loaded chitosan solution neutralized with β-glycerophosphate[32]. Local delivery of paclitaxel from the formulation injected intratumorally was investigated using EMT-6 tumors implanted subcutaneously on Balb/c mice. These experiments showed that one intratumoral injection of the thermosensitive hydrogel containing paclitaxel was as effective as four intravenous injections of Taxol® in inhibiting the growth of EMT-6 cancer cells in mice, but in a less toxic manner.

Synthetic polymers are popular choice mainly for parenteral preparations. The trend in drug delivery technology has been towards biodegradable polymers, requiring no follow up surgical removal, once the drug supply is depleted. Aliphatic polyesters such as poly (lactic acid), poly (glycolic acid), poly (lactide-co-glycolide), poly (decalactone), poly ε-caprolactone have been the subject of the most extensive recent investigations[30]. Various other polymers like triblock polymer systems composed of poly(D,L-lactide)-block-poly(ethylene glycol)-block-poly(DL-lactide), blends of low molecular weight poly(D,L-lactide) and poly(ε-caprolactone) are also in use. These polymers are mainly used for the injectable *in situ* formulations. The feasibility of lactide/glycolide polymers as excipients for the controlled release of bioactive agents is well proven. These materials have been subjected to extensive animal and human trials without evidence of any harmful side effects. When properly prepared under GMP conditions from purified monomers, the polymers exhibit no evidence of inflammatory response or other adverse effects upon implantation.

A thermoplastic triblock copolymer system composed of poly(D,L-lactide)-block-poly(ethylene glycol)-block-poly(D,L-lactide) and blends of low molecular weight poly(D,L-lactide) and poly(∈-caprolactone) (PCL) for the local delivery of taxol in the form of thermoplastic pastes was developed by Zhang *et al*[33]. These dosage forms are injected into the body as a melt and form a semisolid gel mass upon cooling to body temperature. The melting points of these polymeric pastes are greater than 60° therefore the temperature of the paste at the time of injection is at least 60°. This is very painful for the patient and increases the chances of necrosis and scar tissue formation at the site of injection[34]. The other disadvantage is that the release rate of the drug is very slow from these formulations.

An attempt was made to overcome the problem of slow taxol release by using PCL of molecular weight 10-20 KDa as polymeric paste by Dordunoo *et al*[35]. Various water soluble additives such as gelatin, albumin, methylcellulose, dextran and sodium chloride were tried to enhance the taxol release. Taxol and additives were mixed together, pulverized and added in to molten, low molecular weight PCL. *In vivo* drug release and antiangiogenic activity of the *in situ* systems were evaluated using chorio-allantoic membrane. The rate of swelling was observed to be higher with water soluble polymers such as gelatin or albumin. The paste prepared using larger taxol-gelatin particles swelled at a faster rate than those prepared with smaller ones because of water swellable nature of additives used. Such additives when added into polymeric pastes, increased the water imbibitions hence produced a higher rate of drug dissolution and drug release. In chorio-allantoic membrane model, angiogenesis was inhibited. A tumor regression was also observed when *in vivo* efficacy of these composites was investigated using mice tumor model. Another type of synthetic polymeric system includes the *in situ* cross linked system, where the polymers form cross linked networks by means of free radical reactions that may occur by means of light (photopolymerizable systems) or heat (thermosetting systems).

Photopolymerizable systems when introduced to the desired site via injection get photocured *in situ* with the help of fiber optic cables and then release the drug for prolonged period of time[36]. The photo-reactions provide rapid polymerization rates at physiological temperature. Furthermore, the systems are easily placed in complex shaped volumes leading to an implant formation. A photopolymerizable, biodegradable hydrogel as a tissue contacting material and controlled release carrier is reported by Sawhney *et al*[37]. This system consisted of a macromer (PEG-oligoglycolyl-acrylate), a photosensitive initiator (eosin dye) and a light source (UV or visible light). When exposed to light, the system undergoes photopolymerization to form a network. These systems can be used to release water soluble drugs and enzymes at a controlled rate. Argon laser can also be used as a light source. Further investigation of this system for local drug delivery, tissue adhesion prevention, dentistry and orthopaedic applications has been carried out[38–41].

Thermosetting systems are in the sol form when initially constituted, but upon heating[30], they set into their final shape. This sol-gel transition is known as curing. But if this cured polymer is heated further, it may lead to degradation of the polymer. Curing mainly involves the formation of covalent cross links between polymer chains to form a macromolecular network. Dunn *et al*. designed a thermosetting system using biodegradable copolymers of DL-lactide or L-lactide with ε-caprolactone for prosthetic implant and slow release drug delivery systems[42]. This system is liquid outside the body and is capable of being injected by a syringe and needle and once inside the body, it gels.

In *in situ* precipitating polymeric systems, the polymer precipitation from solution may lead to gel formation *in situ* and this precipitation can be induced by change in temperature (thermosensitive systems), solvent removal or by change in pH[43–45]. An important example of thermosensitive polymer is poly-(N-isopropyl acrylamide), [poly (NIPAAM)], which is used for the formation of *in situ* gels. It has lower critical solution temperature phase separation at about 32°[46]. It is unique with respect to the sharpness of its almost discontinuous transition which is usually observed only with ionizable polymers[47]. Pluronics or Poloxamers also undergo *in situ* gelation by temperature change[48,49]. They are triblock copolymers consisting of poly(oxyethylene) and poly(oxypropylene) units that undergo changes in solubility with change in environment temperature.

The polymers such as poly(DL-lactide), poly(DL-lactide-co-glycolide) and poly(DL-lactide-co-∈-caprolactone) form solvent-removal precipitating polymeric systems[50]. When these polymers are dissolved in a water miscible, physiologically compatible solvent such as N-methyl-2-pyrrolidone, propyleneglycol, dimethylsulphoxide, tetrahydrofuran, acetone, 2-pyrrolidone or triacetin and then injected into an aqueous environment, the solvent diffuses into the surrounding aqueous environment while water diffuses into the polymer matrix resulting in a solid polymeric implant.

One of the major problems with these polymers is the possibility of burst effect, during the first few hours after injection into the body. The reason may be attributed to the fact that there is a lag between the injection and formation of the solid implant, as a result burst effect takes place leading to systemic toxicity[30]. The burst effect can be monitored by controlling the molecular weight of the polymer, the solvent used or by adding the surfactant[51,52].

Sucrose acetate isobutyrate (SAIB) is a non crystalline, viscous compound that gets dissolved in some of the organic solvents such as dimethylsulphoxide[53]. SAIB, a sucrose molecule esterified with two acetic acid and six isobutyric acid moieties, is a highly lipophilic, water insoluble sugar and exists as a very viscous liquid. SAIB forms a low viscosity solution when dissolved in organic solvents such as ethanol, NMP, triacetin, and propylene carbonate, which is mixed with active ingredient prior to administration. Once administered, the solvent diffuses out leading to the formation of depot for controlled delivery of active ingredient. The concentration of SAIB, type of solvent, and additives used affect release rate of drug from depot formed *in situ*.

A pH induced *in situ* precipitating polymeric system (an aqueous solution of carbopol-HPMC system) was designed and developed by Ismail *et al.* for plasmid DNA delivery[54]. Mixture of poly (methacrylic acid) and poly (ethylene glycol) dissolved in NMP-ethanol-buffer (1:1:2) was also tried out as polymeric system. It was found that physical stability of pDNA from these systems was not maintained. A high burst effect was also observed with this polymeric system. The reason for less release or no release after burst effect may be attributed to strong interaction between pDNA and polymers used. The removal of the ethanol from the delivery system would have led to a significant reduction in the volume of system thus causing the burst effect.

Ito *et al.* designed and synthesized injectable hydrogels that are formed *in situ* by cross-linking of hydrazide modified hyaluronic acid with aldehyde modified versions of cellulose derivatives such as carboxymethylcellulose, hydroxypropylmethylcellulose and methylcellulose[55]. These *in situ* forming gels were used for preventing postoperative peritoneal adhesions thus avoiding pelvic pain, bowel obstructions and infertility. *In vivo* experiments using rabbit side wall defect-bowel abrasion model, a significant reduction of peritoneal adhesions was observed as compared to that resulting from the administration of saline solution.

## *IN SITU* FORMING NASAL DRUG DELIVERY SYSTEMS

An *in situ* gel system for nasal delivery of mometasone furoate was developed and evaluated for its efficacy for the treatment of allergic rhinitis[56]. Gellan gum and xanthan gum were used as *in situ* gel forming polymers. Animal studies were conducted using an allergic rhinitis model and the effect of *in situ* gel on antigen induced nasal symptoms in sensitized rats was observed. *In situ* gel was found to inhibit the increase in nasal symptoms as compared to marketed formulation nasonex (mometasone furoate suspension 0.05%). Intact ciliated respiratory epithelium and normal goblet cell appearance indicated from histopathology of rat nasal cavity proved that these formulations were safe for nasal administration.

Wu *et al.* designed a new thermosensitive hydrogel by simply mixing N-[(2-hydroxy-3-methyltrimethylammonium)propyl]chitosan chloride and poly (ethylene glycol) with a small amount of α-β-glycerophosphate; for nasal delivery of insulin[57]. The formulation was in solution form at room temperature that transformed to a gel form when kept at 37°. Animal experiments demonstrated hydrogel formulation to decrease the blood-glucose concentration by 40-50% of the initial values for 4-5 h after administration with no apparent cytotoxicity. Therefore, these types of systems are suitable for protein and peptide drug delivery through nasal route. The summary of some reported studies investigating the sustained release of drug from *in situ* gels is depicted in Table 1.

**TABLE 1:** SUMMARY OF SOME REPORTED STUDIES INVESTIGATING THE SUSTAINED RELEASE BY *IN SITU* GELS

Drue	Polymer used	Route of administration	Results*
Theophylline[58]	Gellan gum	Oral	Four-five fold increase of bioavailability in rats and threefold increase in rabbits as compared to commercial oral formulation.
Doxorubicin[59]	Human serum albumin and tartaric acid derivative	Injectable	Sustained delivery of anticancer drug for a long period approx. 100 h.
Testosterone[60]	Poly-lactic acid and PLGA	Injectable	A controlled zero order *in vitro* release was observed.
Pheniramine maleate and albumin FITC[61]	Polyacrylic acid and polymethacrylic acid	Injectable	Sustained delivery of pheniramine for 2d and of albumin-FITC for 5 d.
Recombinant human interleukin-2[62]	Physically cross linked dextran	Injectable	Drug loaded hydrogel releases drug over a period of 5 d. Excellent biodegradability and biocompatibility.
Paracetamol and ambroxol'631	Pectin	Oral	Sustained oral delivery

* All the *in situ* gel forming polymers sustain the release of drug from the delivery system with better bioavailability and more effectiveness. Synthetic polymers exhibit a problem of drug burst release effect.

## EVALUATION AND CHARACTERIZATION OF *IN SITU* GELS SYSTEMS

*In situ* gels may be evaluated and characterized for the following parameters;

### Viscosity and rheology[64]:

This is an important parameter for the *in situ* gels, to be evaluated. Viscosity and rheological properties of *in situ* forming drug delivery systems may be assesses using Brookfield rheometer or some other type of viscometers such as Ostwald's viscometer. The viscosity of these formulations should be such that no difficulties are envisaged during their administration by the patient, especially during parenteral and ocular administration.

### Sol-Gel transition temperature and gelling time:

For *in situ* gel forming systems incorporating thermoreversible polymers, the sol-gel transition temperature may be defined as that temperature at which the phase transition of sol meniscus is first noted when kept in a sample tube at a specific temperature and then heated at a specified rate[11]. Gel formation is indicated by a lack of movement of meniscus on tilting the tube. Gelling time is the time for first detection of gelation as defined above[11].

### Gel Strength:

This parameter can be evaluated using a rheometer. Depending on the mechanism of the gelling of gelling agent used, a specified amount of gel is prepared in a beaker, from the sol form[11]. This gel containing beaker is raised at a certain rate, so pushing a probe slowly through the gel. The changes in the load on the probe can be measured as a function of depth of immersion of the probe below the gel surface.

### *In vitro* drug release studies:

For the *in situ* gel formulations to be administered by oral, ocular or rectal routes, the drug release studies are carried out by using the plastic dialysis cell[7,9]. The cell is made up of two half cells, donor compartment and a receptor compartment. Both half cells are separated with the help of cellulose membrane. The sol form of the formulation is placed in the donor compartment. The assembled cell is then shaken horizontally in an incubator. The total volume of the receptor solution can be removed at intervals and replaced with the fresh media. This receptor solution is analyzed for the drug release using analytical technique. For injectable *in situ* gels[65], the formulation is placed into vials containing receptor media and placed on a shaker water bath at required temperature and oscillations rate. Samples are withdrawn periodically and analyzed.

### Fourier transform infra-red spectroscopy and thermal analysis[64]:

During gelation process, the nature of interacting forces can be evaluated using this technique by employing potassium bromide pellet method. Thermo-gravimetric analysis can be conducted for *in situ* forming polymeric systems to quantitate the percentage of water in hydrogel. Differential scanning calorimetry is used to observe if there are any changes in thermograms as compared with the pure ingredients used thus indicating the interactions.

### Texture analysis[64]:

The firmness, consistency and cohesiveness of hydrogels are assessed using texture analyzer which mainly indicates the syringeability of sol so the formulation can be easily administered *in vivo*. Higher values of adhesiveness of gels are needed to maintain an intimate contact with surfaces like tissues.

## RECENT ADVANCES

One of the challenges facing today's pharmaceutical industry centers on coming up with efficient treatment options that are readily acceptable to physicians and patients. Delivery systems must also contribute to a better therapeutic outcome if they are going to provide viable alternatives to pharmaceuticals currently delivered by other routes. *In situ* gel formulations are one of the challenging drug delivery systems.

Various biodegradable polymers are used for formulation of *in situ* gels, but there are fabrication problems, difficult processability, use of organic solvents for their preparation (especially for synthetic polymer based systems), burst effect and irreproducible drug release kinetics. Natural polymers satisfy the characteristics of an ideal polymer but batch to batch reproducibility is difficult therefore synthetic polymers are used.

Poly(ether-ester) based biodegradable block copolymers are available such as poly(ethylene oxide)-poly(lactic acid) i.e. (PEO-PLA) copolymer, poly(ethyleneoxide)-poly(caprolactone) i.e. (PEO-PCL), poly(ethyleneglycol)-poly(lactide-co-glycolide)-poly(ethylene glycol) i.e. (PEG-PLGA-PEG) copolymer may also be used for formation of *in situ* injectable hydrogels showing improved biocompatibility, biodegradability, reduced burst effect, better mechanical strength and processability[66]

The recent advancement of biotechnologies has led to the development of labile macromolecular therapeutic agents that require complex formulations for their efficient administration[67]. *N*-stearoyl L-alanine(m)ethyl esters when mixed with a vegetable oil and a biocompatible hydrophilic solvent led to the formation of injectable, *in situ*-forming organogel. Following subcutaneous injection, leuprolide-loaded organogel degraded and gradually released leuprolide for 14 to 25d.

## COMMERCIAL FORMULATIONS OF *IN SITU* POLYMERIC SYSTEMS AT A GLANCE

### Timoptic-XE[68]:

It is a timolol maleate ophthalmic gel formulation of Merck and Co. Inc., supplied as a sterile, isotonic, buffered, aqueous gel forming solution of timolol maleate. This formulation is available in two dosage strengths 0.25% and 0.5% in market. The pH of the solution is approximately 7.0, and the osmolarity is 260-330 mOsm. Each ml of Timoptic-XE 0.25% contains 2.5 mg of timolol (3.4 mg of timolol maleate). Inactive ingredients include gellan gum, tromethamine, mannitol, and water for injection and the preservative used is benzododecinium bromide 0.012%. Timoptic-XE, when applied topically on the eye, reduces the elevated, as well as normal intraocular pressure, whether or not accompanied by glaucoma.

### Regel depot technology[69]:

Regel is one of the Macromed's proprietary drug delivery system and based on triblock copolymer, composed of poly (lactide-co-glycolide)-poly (ethylene glycol)-poly(lactide-co-glycolide). It is a family of thermally reversible gelling polymers developed for parenteral delivery that offers a range of gelation temperature, degradation rates and release characteristics as a function of molecular weight, degree of hydrophobicity and polymer concentration. Following injection, the physical properties of polymer undergo a reversible phase change resulting in formation of a water insoluble, biodegradable gel depot. Oncogel® is a frozen formulation of paclitaxel in Regel. It is a free flowing liquid below room temperature which upon injection forms a gel *in situ* in response to body temperature. hGHD-1 is a novel injectable depot formulation of human growth hormone (hGH) utilizing Macromed's Regel drug delivery system for treatment of patients with hGH deficiency.

### Cytoryn[70,71]:

This is one of the Macromed's products, which is a novel, peritumoral, injectable depot formulation of interleukin-2 (IL-2) for cancer immunotherapy using Regel drug delivery system. It is a free flowing liquid below room temperature that instantly forms a gel depot upon injection from which the drug is released in a controlled manner. Cytoryn enhances the immunological response by safely delivering four times the maximum tolerated dose allowed by conventional IL-2 therapy. Cytoryn also activates the systemic antitumor immunity. Regel system stabilizes and releases IL-2 in its bioactive form. The release of drugs is controlled by the rate of diffusion from and degradation of the depot.

## CONCLUSION

In conclusion, the primary requirement of a successful controlled release product focuses on increasing patient compliance which the *in situ* gels offer. Exploitation of polymeric *in situ* gels for controlled release of various drugs provides a number of advantages over conventional dosage forms. Sustained and prolonged release of the drug, good stability and biocompatibility characteristics make the *in situ* gel dosage forms very reliable. Use of biodegradable and water soluble polymers for the *in situ* gel formulations can make them more acceptable and excellent drug delivery systems.
